# Phosphocreatine Interacts with Phospholipids, Affects Membrane Properties and Exerts Membrane-Protective Effects

**DOI:** 10.1371/journal.pone.0043178

**Published:** 2012-08-17

**Authors:** Malgorzata Tokarska-Schlattner, Raquel F. Epand, Flurina Meiler, Giorgia Zandomeneghi, Dietbert Neumann, Hans R. Widmer, Beat H. Meier, Richard M. Epand, Valdur Saks, Theo Wallimann, Uwe Schlattner

**Affiliations:** 1 University Joseph Fourier, Laboratory of Fundamental and Applied Bioenergetics (LBFA), Grenoble, France; 2 Inserm, U1055, Grenoble, France; 3 Department of Biochemistry and Biomedical Sciences, McMaster University, Hamilton, Canada; 4 Institute of Cell Biology, ETH Zurich, Zürich, Switzerland; 5 Institute of Physical Chemistry, ETH Zurich, Zürich, Switzerland; 6 Department of Neurosurgery, University of Berne, Inselspital, Berne, Switzerland; University of Cambridge, United Kingdom

## Abstract

A broad spectrum of beneficial effects has been ascribed to creatine (Cr), phosphocreatine (PCr) and their cyclic analogues cyclo-(cCr) and phospho-cyclocreatine (PcCr). Cr is widely used as nutritional supplement in sports and increasingly also as adjuvant treatment for pathologies such as myopathies and a plethora of neurodegenerative diseases. Additionally, Cr and its cyclic analogues have been proposed for anti-cancer treatment. The mechanisms involved in these pleiotropic effects are still controversial and far from being understood. The reversible conversion of Cr and ATP into PCr and ADP by creatine kinase, generating highly diffusible PCr energy reserves, is certainly an important element. However, some protective effects of Cr and analogues cannot be satisfactorily explained solely by effects on the cellular energy state. Here we used mainly liposome model systems to provide evidence for interaction of PCr and PcCr with different zwitterionic phospholipids by applying four independent, complementary biochemical and biophysical assays: (i) chemical binding assay, (ii) surface plasmon resonance spectroscopy (SPR), (iii) solid-state ^31^P-NMR, and (iv) differential scanning calorimetry (DSC). SPR revealed low affinity PCr/phospholipid interaction that additionally induced changes in liposome shape as indicated by NMR and SPR. Additionally, DSC revealed evidence for membrane packing effects by PCr, as seen by altered lipid phase transition. Finally, PCr efficiently protected against membrane permeabilization in two different model systems: liposome-permeabilization by the membrane-active peptide melittin, and erythrocyte hemolysis by the oxidative drug doxorubicin, hypoosmotic stress or the mild detergent saponin. These findings suggest a new molecular basis for non-energy related functions of PCr and its cyclic analogue. PCr/phospholipid interaction and alteration of membrane structure may not only protect cellular membranes against various insults, but could have more general implications for many physiological membrane-related functions that are relevant for health and disease.

## Introduction

The guanidino compounds creatine (Cr) and phosphocreatine (PCr) belong to the most abundant cellular metabolites in mammals including humans, with about 120 g present in a 70 kg adult male [1]. Tissues such as muscle or brain maintain a total cellular Cr pool of up to 30–40 mM [1]. Cr exerts a large number of pleiotropic beneficial physiological and pharmacological effects *in vitro* and *in vivo*. These include protective effects against hypoxic, ischemic, oxidative, neurodegenerative or muscular damage, and possibly also beneficial effects for healthy subjects in respect to life expectancy and life quality, learning and memory or during pregnancy (reviewed in [1]–[6]). The beneficial effects of Cr supplementation were first reported for muscle growth, performance and rehabilitation in sports [7]–[10]. More recently, protective effects were observed in animal models of various pathologies and also in some clinical studies [11]–[14], proposing Cr supplementation as a valuable clinical strategy for muscle and bone growth and maintenance, as well as for general cell- and neuroprotection (reviewed in [5], [15], [16]).

About 50% of the daily requirement of Cr is obtained by endogenous synthesis in the body, mainly in kidney and liver, the remainder comes from nutrition, principally fish and meat. Cr is taken up into cells by a specific Cr transporter [4], [17]. In cells, the isoforms of creatine kinase (CK) reversibly convert about two thirds of the total Cr pool into PCr. With intracellular concentrations up to 30–40 mM and its rapid diffusibility, PCr together with CK isoforms provides an efficient energy buffer and transport system that maintains cellular energy homeostasis by restoring global and local ATP pools [5], [16], [18]. Thus, most Cr is found in cells with high and variable energy requirements such as myocytes or neurons. Some evidence suggests that beneficial effects of Cr depend on the presence of CK and increased PCr generation [4], [19], [20].

Energy-related functions of the CK/PCr system may well explain the ergogenic effects of Cr on muscle performance in sports and clinics [10], but may be insufficient for other pleiotropic Cr effects observed in complex pathologies like neurodegenerative disease or aging, including altered cell signaling, protein expression and cell protection in general [9], [10], [21], [22]. In a *Drosophila* model, it was shown that Cr alone can protect from oxidative stress, although insects, expressing arginine kinase instead or CK, are not capable to synthesize PCr [23], [24]. Mechanistic aspects were also raised for effects observed with synthetic Cr analogues, in particular cyclocreatine (cCr). An anti-tumor activity of cCr observed *in vitro* [25]–[27] and in transplanted tumors [28], [29] was initially explained by energy depletion due to trapping of high energy phosphates via the CK reaction into PcCr, a poor substrate in the CK reverse reaction [30]. However, follow-up studies showed that Cr itself also displays anti-tumor activity [29], [31] and that Cr and cCr can have beneficial effects in neuroprotection [32] and as a potential treatment for cognitive dysfunctions in Cr transporter deficiency [33], respectively. These data rather exclude purely energy-related mechanisms of Cr and congeners, at least in these studies.

Different alternative mechanisms for Cr action have already been proposed. Mitochondrial CK could mediate anti-oxidative or anti-apoptotic effects by maintaining local substrate cycling with inhibitory effects on mitochondrial ROS production and pro-apoptotic permeability transition [34]–[36]. However, mitochondrial CK seems to be dispensable for at least some protective effects [37]. Cr was further proposed to exert direct antioxidant effects [38]–[41], activate signaling pathways like Akt/PKB [9], [10], [21], [42] or AMPK [43], leading to increased expression of muscle transcription factors [9], [10], [21] and enzymes for oxidative stress defense [22]. However, Cr *in vitro* is not an effective antioxidant, and Cr may not directly affect cell signaling, as e.g. direct activation of AMPK by PCr/Cr ratios [44] has been questioned [45], [46].

Here, we propose a novel direct molecular mechanism of Cr action, involving molecular interaction mainly of PCr with membrane phospholipids. This leads to membrane protection and stabilization, possibly affecting additional membrane-based processes like ion homeostasis and cell signaling. Earlier observations showed that exogenous PCr in cardioplegic solution is cardioprotective, although external PCr is unlikely to transverse cardiac plasma membranes [47]. A possible mechanism could be inferred, however, from ESR data that already suggested that PCr may be able to interact with membranes and to increase membrane packing [48], [49]. In support of this notion, more recent proton NMR studies on human muscle tissue suggested a motionally restricted Cr/PCr pool bound to cellular structures, possibly phospholipids of cellular membranes [50], [51].

In the present study, we directly tested lipid interaction of both, Cr and PCr, as well as their cyclic analogues cCr and PcCr, using independent biochemical and biophysical assays. The results demonstrate a low affinity interaction in particular of the phospho-compounds, PCr and PcCr, with zwitterionic phospholipids. They further reveal changes in phospholipid bilayer properties by membrane-bound PCr, and a resulting protection of lipid membranes against permeabilization and cell lysis. These findings propose a novel mechanism for the biological functions of Cr and PCr acting at membranes, in addition to the known energy-related functions, both with relevance for human health and disease.

## Materials and Methods

### Materials

Cr was from Sigma (Munich, Germany), PCr from Calbiochem (La Jolla, CA, USA), cCr and PcCr from Avicena (Cambridge, MA, USA), glucose-6-phosphate from Applichem (Darmstadt, Germany). If not stated otherwise, chemicals were from Sigma-Aldrich (Buchs, Switzerland).

### Large Unilamellar Vesicles (LUVs)

Lipid stock suspensions at 5 mg/ml in 10 mM TES, pH 7.0, 50 mM NaCl were prepared as described [52]. If not stated otherwise, the following mixtures of purified lipids were used: (i) a composition mimicking the plasma membrane containing 30.2% (w/w) cholesterol (CH; Sigma-Aldrich), 23.7% (w/w) phosphatidylethanolamine (PE; from egg yolk), 23.7% (w/w) phosphatidylcholine (PC, lecithin; from egg yolk) and 22.4% (w/w) sphingomyelin (SP; from bovine spinal cord; all Grade 1 from Lipid Products, South Nutfield, Great Britain); (ii) a composition mimicking the mitochondrial inner membrane (PC/CL) containing 84% (w/w) PC (Lipid Products) and 16% (w/w) cardiolipin (CL; Fluka, Buchs, Switzerland). For SPR experiments, 0.1% (w/w) *N*-((6-(Biotinoyl)amino)hexanoyl)-1,2-dihexadecanoyl-PE (Biotin-X-DHPE; Molecular Probes, Leiden, Netherlands) was added. LUVs with a diameter of about 160 nm were prepared from the lipid stock suspension by a combination of freeze/thawing and extrusion techniques [52], stored at 4°C and used within 4 days. The quality of LUVs was routinely checked by electron microscopy. Preparation of LUVs for leakage assays is described below.

### Vesicle Retention Assay with Chemical Creatine Determination

Aliquots of LUV suspensions were incubated for 6 min at room temperature with Cr or PCr at 0, 10, 30, and 100 mM in 10 mM TES, pH 7.0, 50 mM NaCl. Note that Cr can be used only up to 100 mM due to its solubility limit. After centrifugation for 30 min at 4°C and 15000×g, the pellet was washed twice with 1 ml of buffer. To hydrolyze PCr to Cr, 0.1 M HCl was added to the pellet, incubated for 15 min at 90°C, cooled down to room temperature, and neutralized with 0.1 M NaOH. Cr in hydrolyzed and non-hydrolyzed samples was determined by diacetyl-αlpha-napthol reaction according to [53].

### Surface Plasmon Resonance Spectroscopy (SPR)

Binding analysis of Cr, PCr, glucose and analogues to model lipid LUVs was performed by SPR with a T100 Biacore instrument (GE Healthcare, France). For each programmed series of measurement cycles, the four lanes of a CM5 sensor chip were coated with streptavidin (Sigma-Aldrich) and either used to immobilize 1500 RU biotinylated LUVs (CH/PE/PC/SP or PC/CL) or left empty (controls) as described [52], [54]. Each measurement cycle (immobilisation of LUVs, contact phase and dissociation phase of 240 s each, surface regeneration) was performed with running buffer (10 mM TES, pH 7.0, 50 mM NaCl) at a flow rate of 10 µl/min and 25°C. Metabolites (Cr, PCr, glucose or analogues) were injected into the flow over all four lanes of the sensor chip. After dissociation, the original baseline was recovered by injecting 100 mM NaCl for 10 s and an additional washing step. For each series of measurement cycles, the surface was regenerated with 0.5% SDS to remove LUVs. Binding was determined as difference signal between LUV-covered and empty streptavidin surfaces and quantified by the SPR signal remaining after 60 s of dissociation. This is preferable to avoid potential problems due to interference of strong bulk refractive index changes at high metabolite concentrations during association phase. Since the SPR signal depends not only on the number but also on the molecular mass of the bound analyte, SPR data were expressed as response units (RU) divided by the molecular mass (Da) of the corresponding analyte.

### NMR Measurements

For NMR experiments, a CH/PE/PC/SP lipid suspension (150 mg/ml in 10 mM TES, pH 6) containing 30.2% (w/w) CH, 23.7% (w/w) dimyristoyl PE (DMPE), 23.7% (w/w) dimyristoyl PC (DMPC), and 22.4% (w/w) bovine brain SP (all from Avanti Polar Lipids, Alabaster, AL, USA) were mixed with freshly prepared solutions of Cr, PCr or glucose (final concentrations: 75 mg/ml for lipids, 25 mM for Cr, PCr, and glucose), or equivalent volume of TES buffer alone (control). This suspension containing spontaneously formed vesicles was submitted to five cycles of freezing, thawing and vortexing, resulting in the formation of multilamellar vesicles (MLV). The suspension was centrifuged (10 min, 24000×g, 30°C) and the pellets were transferred into Chemagnetics 4 mm NMR rotors. Experiments were performed at a magnetic field of 7.0 Tesla with a Varian Infinity+ spectrometer and a double resonance Chemagnetics MAS probehead. In the static ^31^P NMR experiments the length of the 90° pulses was 6.3 µs, the spectral width 50 kHz, the recycle delay 3 s. Continuous wave ^1^H decoupling was employed with a ^1^H decoupling strength of 30 kHz. All experiments were performed with freshly prepared samples.

### Differential Scanning Calorimetry (DSC)

DSC runs were performed using 3 mM 1-stearoyl-2-oleoyl PC (SOPC; Avanti Polar Lipids) suspensions. Lipid films were hydrated with either buffer alone (20 mM PIPES pH 7.4, 1 mM EDTA, 140 mM NaCl) or with added 100 mM PCr, Cr or *N,N-*dimethylbiguanidinium chloride (DMBG; Sigma, Canada). In additional experiments, either NaCl was replaced by an equivalent concentration of guanidine-HCl, or 140 mM NaCl/100 mM PCr (disodium salt) were replaced by 500 mM PCr (disodium salt) or by PCr (di(Tris) salt) without additional salt. Thermograms were obtained by heating-cooling cycles between 0 and 15°C. The shifts in temperature with respect to SOPC were recorded for two successive heating-cooling cycles. The experiments were repeated three times and the transition temperatures averaged.

### Leakage Assay

LUVs (100 nm in diameter) consisting of 30.2% CH, 23.7% 1- palmitoyl-2-oleoyl PE (POPE), 22.4% 1-palmitoyl-2-oleoyl PC (POPC), and 23.7% bovine brain SP (all from Avanti Polar Lipids) were generated by extrusion, entrapping at the same time a mixture of 8-aminonaphthalene-1,3,6-trisulfonate (ANTS) and the cationic quencher p-xylene-bis-pyridinium bromide (DPX; both from Invitrogen, Canada). Fifty µM of LUVs were then placed in buffer alone (20 mM PIPES pH 7.4, 1 mM EDTA, 140 mM NaCl) or in buffer containing 100 mM PCr, Cr or DMBG, with osmolarity of all solutions adjusted to the same value as the buffer itself and that of the ANTS/DPX that was entrapped in the vesicles (about 320 mOsm). Leakage was induced with 500 nM melittin (Sigma, Canada). Fluorescence dequenching of the ANTS/DPX mixture due to leakage into solution was monitored at 360 nm excitation/530 nm emission for 200 s, before full permeabilization of LUVs with 20% Luberol PX to a final concentration of 0.1% in the cuvette. Each compound was tested twice.

### Red Blood Cell Hemolysis Assay

Blood from a freshly slaughtered pig obtained from the local slaughterhouse (Abattoir de Grenoble, France) was mixed with an anticoagulant (citrate buffer) and centrifuged for 15 min at 1500×g at room temperature. The pellet consisting of red blood cells (RBC) was washed four times with phosphate buffered saline (containing 140 mM NaCl, 1.5 mM KH_2_PO_4_, 7.5 mM Na_2_HPO_4_ and 10 mM glucose). The final pellet was resuspended in phosphate buffered saline, stored at 4°C and used for assays within 2–3 days. For assays, the concentration of RBC was adjusted to the concentration yielding 1.97–2.00 OD (575 nm) after full hemolysis induced by distilled water. In the hemolysis assay, RBC suspension in buffer (30 mM Hepes pH 7.4 for doxorubicin or hypoosmotic stress assay, phosphate buffered saline for saponin assay) was preincubated for 10 min under constant shaking without or with 50 mM PCr, glucose or glucose-6-phosphate (Glc-6-P); osmolarity was adjusted with NaCl to ca. 330 mOsm. Then, lysing agent was added (1–10 µg/ml saponin, 300 µM doxorubicin; both from Sigma-Aldrich) or for hypoosmotic stress, the preincubated RBC were placed in hyposmotic medium (200 mOsm) and incubated (in presence of the tested metabolites) on a shaker at room temperature (saponin, hypoosmotic stress) or 37°C (doxorubicin) for time periods of 5 h (doxorubicin) or 10 min (saponin, hypoosmotic stress). After centrifugation of samples for 20 s at 15000×g, hemoglobin concentration in the supernatant was measured spectrophotometrically at 575 nm. For each blood sample, results were normalized to full hemolysis induced by resuspension in distilled water. The stability of PCr under the given conditions during the entire experiment was checked by HPLC.

### Molecular Model

Representations of molecular structures were prepared in WebLabViewer Pro v4.0. PDB files for Cr and PCr were from the human metabolome database (http://www.hmdb.ca), accession numbers HMDB00064 (Cr) and HMDB01511 (PCr). The PDB file for the eight dioleoyl PC (DOPC) phospholipids is a subset of a molecular dynamics simulation of 200 DOPC molecules in the gel phase, published by Heller and colleagues [55] (GEL.PDB available http://www.lrz.de/~heller/membrane/membrane.html). The solvent-accessible surface area of Cr and PCr was calculated by WebLab Viewer Pro 4.0 using a probe radius of 1.4 Å. PCr was manually docked onto a part of the modeled DOPC surface consisting of eight phospholipids.

## Results

### Phosphocreatine and Creatine Both Co-sediment with Liposomes

**Figure 1:**
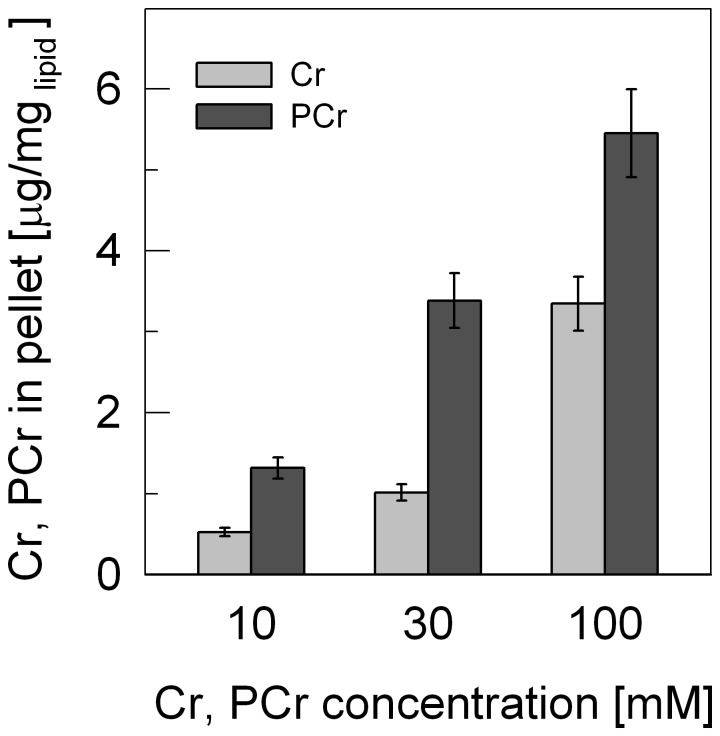
Phosphocreatine and creatine are retained by liposomes as determined in a biochemical assay. LUVs consisting of phosphatidylcholine and cardiolipin (PC/CL) were incubated either with Cr or PCr at different concentrations in TES buffer (10 mM pH 7.0, 50 mM NaCl), centrifuged, the pellet washed twice, and remaining Cr and PCr quantified. Data are given as mean±SD (n = 3).

Liposomes in form of LUVs consisting of 84% phosphatidylcholine (PC) and 16% cardiolipin (CL), which mimic the mitochondrial inner membrane, have been used in several of our earlier studies to analyze binding of mitochondrial kinases to such membranes by surface plasmon resonance (SPR) [52], [56], [57]. When we included substrates of CK at physiological concentrations (10–30 mM), dissociation profiles indicated low level binding of PCr (not shown). To confirm such observations, the same LUVs were incubated with Cr or PCr, washed twice and subjected to biochemical quantification (Fig. 1). The data showed concentration-dependent association of Cr and PCr with the PC-CL liposomes that was much stronger with PCr compared to Cr.

### Phosphocreatine and Analogues Bind Phospholipids in Surface Plasmon Resonance

**Figure 2:**
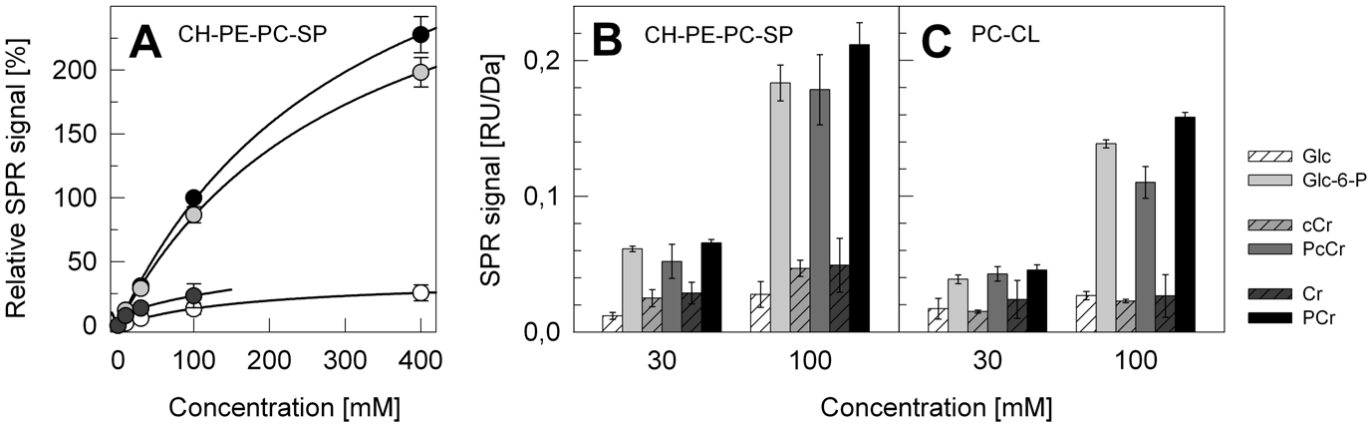
Direct interaction of phosphocreatine, creatine, their cyclic analogues, glucose and glucose phosphate with liposomes as determined by surface plasmon resonance. (A) Concentration-dependence of glucose (Glc, white), Cr (dark grey), glucose-6-phosphate (Glc-6-P, light grey), and PCr (black) binding to LUVs consisting of cholesterol, phosphatidylethanolamine, phosphatidylcholine, and sphingomyelin (CH/PE/PC/SP). (B, C) Binding of PCr, Cr, their cyclic analogues, as well as glucose and Glc-6-P to LUVs consisting of either (B) CH/PE/PC/SP or (C) PC/CL. Runs were performed in TES buffer (10 mM pH 7.0, 50 mM NaCl). Bound metabolites were quantified at a reporting point 60 s after beginning of dissociation. The given SPR signal corresponds to measured response units (RU) divided by the molecular mass (Da) of the corresponding metabolite. Data in (A) were normalized to the response at 100 mM PCr. All data are given as mean ± SD (n = 4 independent experiments).

In the following, the interaction of Cr and PCr, but also of their synthetic cyclic analogues cCr and PcCr as well as glucose and Glc-6-P as controls were analyzed more systematically. We used an SPR application developed in our earlier studies on protein/membrane interactions [52], [54], [56]–[58] as a direct and quantitative biophysical method. The PC/CL-LUVs as above were compared with plasma membrane-like LUVs consisting of cholesterol, phosphatidylethanolamine, phosphatidylcholine and sphingomyelin (CH/PE/PC/SP). SPR measurements of low affinity interactions requiring high concentrations of injected binding partner can only be performed during dissociation phase; we chose a reporting point after 60 s of dissociation (Fig. 2). The SPR data provide first direct evidence for concentration-dependent, saturable PCr binding to CH/PE/PC/SP-LUVs that was slightly higher than with Glc-6-P and much higher as compared to Cr or glucose. From the concentration-dependence of binding, a K_D_ for PCr can be estimated to be around 100 mM (Fig. 2A). However, all compounds with a phosphate moiety showed interaction with both types of membrane in the SPR assay (Fig. 2B,C). PCr also bound to pure PC-LUVs, albeit much weaker (not shown). Finally, it is to note that the SPR response of PCr (and Glc-6-P) of up to 40 RU (after 60 s of dissociation and at 100 mM PCr) is remarkable for such a low Mr compounds. Extrapolated to end association levels, this would correspond to more PCr molecules than could be accommodated in a single layer at the LUV surface. However, the SPR response is also a function of the distance of bound material from the biosensor surface. Thus, a change in LUV shape, compacting the liposome towards the surface, could also explain the high SPR response.

### Creatine and Phosphocreatine Induce Changes in ^31^P-NMR Lipid Spectra

Further studies were limited to PCr and Cr as the archetype guanidino compounds. Interaction of PCr and Cr with CH/PE/PC/SP membranes was further analyzed by solid-state ^31^P-NMR, using multilamellar vesicles (MLVs).

**Figure 3:**
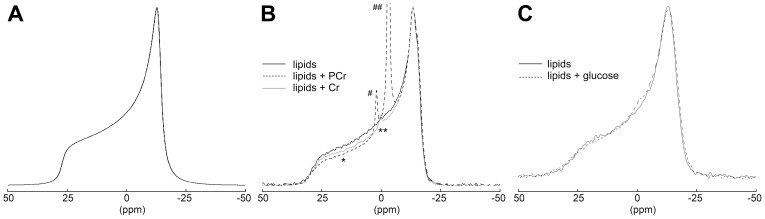
Interaction of phosphocreatine and creatine with lipid vesicles as visualized by solid-state ^31^P-NMR spectroscopy. (A) Simulation of the static ^31^P-NMR spectrum of a spherical liposome. (B), (C) Static spectra of lipid vesicles (CH/PE/PC/SP) incubated with (B) 25 mM Cr or PCr, or (C) 25 mM glucose were compared to spectra of control MLVs without additions. Spectra were recorded at 300 MHz and 303 K, referenced to 85% H_3_PO_4_ and normalized. Differences between control and treated lipid vesicles observed in (B) but not in (C) are indicated: (*) change in the lineshape in the powder spectrum of Cr and PCr; (**) small resonance peak at ∼0 ppm in the Cr spectrum due to isotropic lipids (micelles or other small aggregates); (#) resonance peak at 1.9 ppm in the PCr spectrum corresponding to inorganic phosphate; (##) resonance peak at −3.2 ppm in the PCr spectrum corresponding to the phosphate moiety of PCr.

The ^31^P-NMR lineshape of phospholipid liposomes is determined by the chemical shielding tensor and by the orientation of the phospholipid molecules with respect to the static magnetic field B_0_. In the bilayers in the liquid-crystalline phase the phospholipid molecules undergo fast (in the NMR time scale) rotation about the bilayer normal which partially averages the chemical shielding tensor, resulting in an axially symmetric tensor. The characteristic ^31^P NMR spectrum of a spherical liposome is an axially symmetric powder pattern with a high-field peak and a low-field shoulder, corresponding to the phospholipids oriented with the long axis perpendicular and parallel to B_0_, respectively (Fig. 3A). A preferential orientation of the phospholipids with the normal perpendicular to B_0_ would alter such lineshape, in particular with a reduced intensity of the low field shoulder [59]. We have detected such effect on the ^31^P-NMR signal of phospholipid MLV upon addition of PCr and, to less extent, of Cr (Fig. 3B). In fact, the spectra in Fig. 3 B show a decrease in the low intensity edge relative to the high intensity edge at -13.5 ppm (* in Fig. 3B). The control experiment where glucose was added to the MLV did not show any effect (Fig. 3C).

It can be concluded that PCr (and possibly much less so Cr) interact with the lipid bilayers and might induce a preferential orientation of the phospholipid bilayer with the normal perpendicular to the magnetic field. In addition, the Cr-spectrum reveals a narrow peak at 0 ppm (** in Fig. 3B) that is missing in other spectra. The resonance at 0 ppm corresponds to the isotropic chemical shift of phospholipids and indicates the presence of fast-tumbling lipid aggregates, possibly due to the occurrence of small micelles. Finally, the PCr-spectrum shows the typical narrow peaks due to inorganic phosphate generated by minor PCr hydrolysis (1.9 ppm) and the PCr itself (−3.2 ppm).

### Phosphocreatine Changes the Phase Transition Properties of Lipids

**Figure 4:**
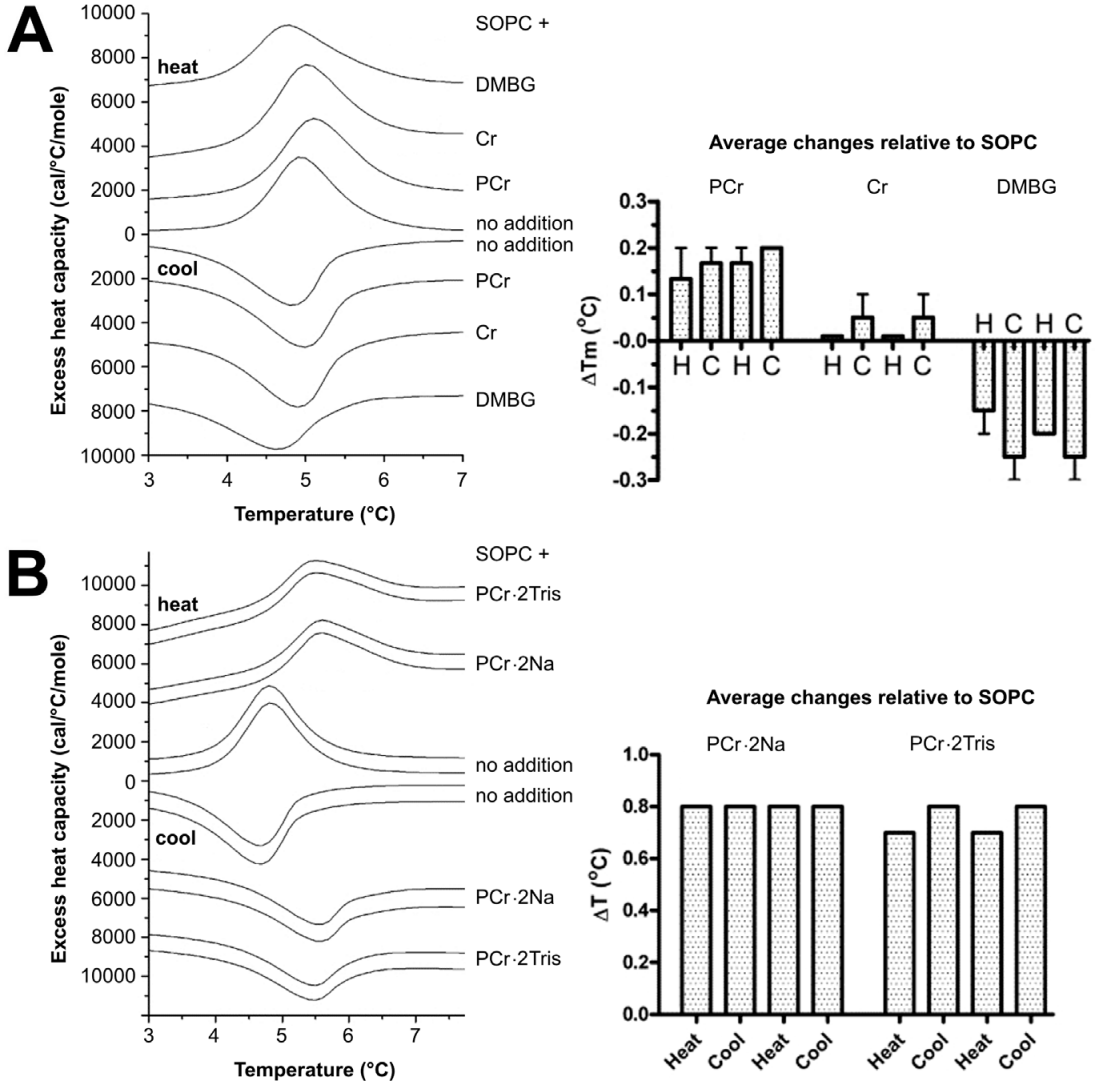
Effect of phosphocreatine, creatine and dimethylbiguanidium chloride on the lipid phase transition as determined by differential scanning calorimetry. Thermograms (left) and quantified phase transition temperature shifts (right) obtained by heating or cooling of a suspension of SOPC in PIPES buffer (20 mM PIPES pH 7.4, 140 mM NaCl, 1 mM EDTA) alone (middle scans in A or B, labeled (−). (A) one heating and one cooling scan shown with added 100 mM PCr, Cr or dimethylbiguanidium chloride (DMBG), or (B) two cycles of heating and cooling shown for each sample. 500 mM PCr Na-salt or Tris-salt (omitting NaCl). Thermograms (left) are from representative runs, histograms (right) represent averaged data from 3 independent experiments (H  =  heating; C  =  cooling).

Next we were interested in analyzing the effect of PCr and Cr binding on lipid membrane properties. We tested the ability PCr, Cr and DMBG (metformin) to change the phase transition properties of stearoyloleoyl phosphatidylcholine (SOPC). This lipid has a gel to liquid crystalline phase transition at ∼5°C, allowing compounds to be tested for their effects on the phase transition without heating to high temperatures. As shown in Fig. 4A, PCr raised the temperature of phase transition and thus stabilized the gel phase of SOPC relative to the liquid crystalline phase. Cr had no effect (or a small positive ΔT_m_) and DMBG had a negative ΔT_m_. To test if the effect was related to salting-out/salting-in effects, we also used guanidine hydrochloride in place of NaCl, and obtained an increase in the phase transition temperature with PCr, showing that there was little effect of the anion (data not shown).

Since the temperature shifts in the experiment shown in Fig. 4A were small although well reproducible, we repeated these experiments at a higher concentration of PCr (500 mM) and compared the PCr-Na salt used throughout this study with the Tris salt, omitting any other cations as NaCl. As expected, this led to higher temperature shifts (Fig. 4B), and again no effect of the cation could be observed. Therefore, the observed shifts in phase transition temperature were due to interaction of PCr with the lipid and not to a solvent-type effect.

### Membrane-stabilizing Effects

**Figure 5:**
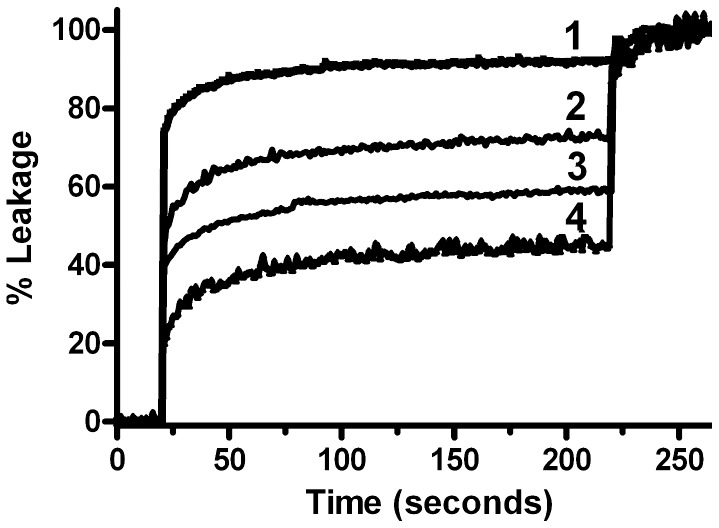
Phosphocreatine and creatine protect lipid vesicles against melittin-induced permeabilization. 50 µM of CH/POPE/POPC/SP-LUVs entrapping a quenched ANTS/DPX solution were injected into PIPES buffer (20 mM pH 7.4, isomolarity of the entrapped solution was adjusted to be equal to that of the external media with NaCl). The media external to the liposomes contained only phosphate buffer and NaCl (trace 1) or 100 mM DMBG (trace 2), Cr (trace 3), or PCr (trace 4). Leakage was begun with 500 nM melittin. All buffers and entrapped ANTS/DPX solution were adjusted to the same osmolarity and each compound was tested twice. Fluorescence dequenching was followed at 530 nm and normalized to the signal obtained after full permeabilization of LUVs.

**Figure 6:**
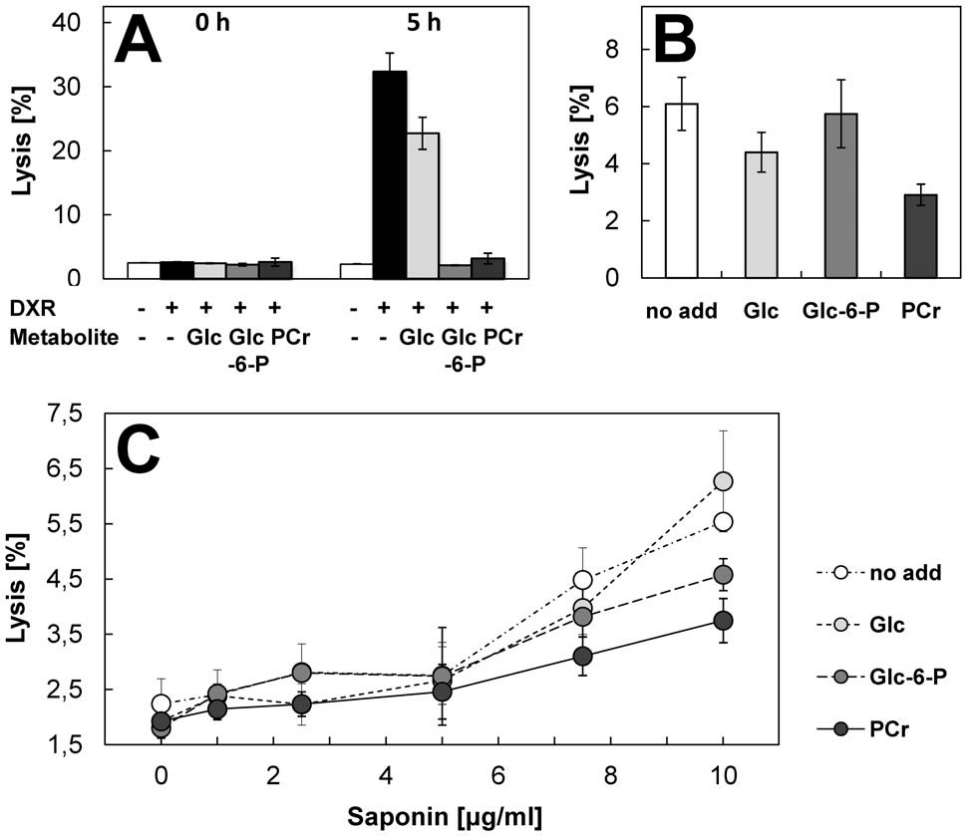
Phosphocreatine protects red blood cells against doxorubicin-, saponin- and hypoosmotic stress- induced lysis. Red blood cells isolated from fresh pig blood were preincubated for 10 min without any additions or in presence of 50 mM PCr, glucose (Glc) or glucose-6-phosphate (Glc-6-P). Hemolysis was induced by (A) 300 µM doxorubicin (DXR) at 37°C, (B) incubation for 10 min at 200 mOsm at room temperature, or (C) incubation with 1–10 µg/ml saponin for 10 min at room temperature. Data in (A) were corrected for absorbance of doxorubicin; hemolysis was evaluated directly (0 h) and 5 h after addition of doxorubicin. Stability of PCr under the given experimental condition was verified by HPLC. Data are given as mean±SD (n = 3). Note: Glc-6-P also exerts a protective effect.

To further study consequences of PCr binding on lipid membrane properties, we used strong membrane-permeabilizing treatments to challenge two very different membrane systems, model lipid vesicles and intact cells. First, CH/POPE/POPC/SP-LUVs were loaded with a mixture of ANTS and DPX at concentrations at which the ANTS fluorescence is quenched. These liposomes were then subjected to a standard leakage assay using the peptide melittin as a pore forming agent. PCr, and to smaller extent also Cr and less so DMBG, prevented permeabilization of LUVs by melittin (Fig. 5). Second, we used a classical hemolysis assay with red blood cells (RBC) to demonstrate that PCr protects RBC from noxious insults by doxorubicin (Fig. 6A), hypoosmotic stress (Fig. 6B) and saponin (Fig. 6C). Hemolysis induced by doxorubicin can serve as a model of membrane damage related to oxidative stress [60], [61]. Supraclinical doxorubicin concentration of 300 µM induced hemolysis over a 5 h observation period. PCr significantly inhibited hemolysis during this time period (Fig. 6A). PCr also reduced hemolysis induced by hypoosmotic stress and the mild detergent saponin (Fig. 6B,C). Protection from membrane permeabilization was also exerted, to variable degree, by Glc-6-P, which indicates that for the observed protective effects, as with the observed membrane interaction, the phosphate moiety plays an important albeit not exclusive role.

## Discussion

The pleiotropic beneficial effects of Cr supplementation are mainly thought to arise from the increased synthesis of the high energy intermediate PCr, thus improving the cellular PCr/ATP ratio energy status, while anti-cancer effects of synthetic cCr were proposed to be based on inhibition of CK-mediated PCr synthesis [1]–[6]. Cr or cCr uptake and intra-cellular accumulation, and probably also conversion to the phosphorylated compounds, seem to be a prerequisite for most effects of Cr or cCr supplementation, but an exclusive bioenergetic function of the CK/PCr system has been questioned. Many beneficial effects of Cr and analogues that are linked to cell signaling and cell protection [9], [10], [21], [22] do not relate to bioenergetics, and some studies directly challenged an involvement of bioenergetics [32]. Among the proposed additional functions are direct anti-oxidative effects of Cr [39] and membrane stabilization by PCr [49]. However, the anti-oxidative capacity of Cr *in vitro* is low, and mechanistic, quantitative evidence for membrane interaction has been scarce. In the present study, several independent biochemical and biophysical approaches including chemical detection, SPR, ^31^P-NMR, and DSC were applied to resolve this issue. All methods consistently demonstrated that PCr can indeed (i) directly bind to phospholipid-containing membranes albeit with low affinity, (ii) alter structural and conformational parameters of phospholipid liposomes, and (iii) protect phospholipid liposomes and erythrocytes from permeabilization induced by melittin, doxorubicin, hypoosmotic stress or saponin.

Binding of Cr, PCr and analogues to model membranes mimicking the lipid composition of the plasma membrane and the inner mitochondrial membrane was quantified biochemically and more extensively by surface plasmon resonance spectroscopy. High binding levels were observed for the phosphorylated species (PCr, PcCr, and also Glc-6-P). More significant effects of the phosphorylated form of Cr have been also observed in earlier studies on preservation of organ transplants when added to cardioplegic solutions [47], [49] and on protection from oxidative damage [62]. Since the PCr infused with cardioplegic solutions is unlikely to transverse biomembranes, due to its charge, it has to be assumed that it will bind to the outer leaflet of cellular plasma membranes in a similar fashion as intracellular PCr formed by CK from Cr would interact with the inner leaflet of the same membrane. Our data further show that the linear and cyclic guanidino compounds examined here behave similarly in respect to their membrane binding capacity. This is quite in contrast to their function as CK substrates, where Cr/PCr allow reversible catalysis, while cCr is rather trapped into PcCr [30]. However, this observation would be consistent with similar anticancer effects observed for Cr and cCr [29], [31].

Membrane interaction of PCr has important physiological significance, even if it is shared to a certain degree by other phospho-compounds like Glc-6-P. As a product of the CK reaction, PCr accumulates within the cell to high concentrations, reaching several tens of mM in tissues like muscle [1], and as such it is one of the most abundant metabolites. These concentrations would be relevant for phospholipid interaction, since our SPR results suggest a K_D_ of in the higher mM-range. Concentrations of other phospho-compounds are much lower, with Glc-6-P normally not exceeding 1 mM [63], [64]. Interestingly, however, even for Glc-6-P some membrane-shielding effects have been suggested e.g. in the protection against alpha-chaconine-induced developmental toxicity in Xenopus embryos [65].

**Figure 7:**
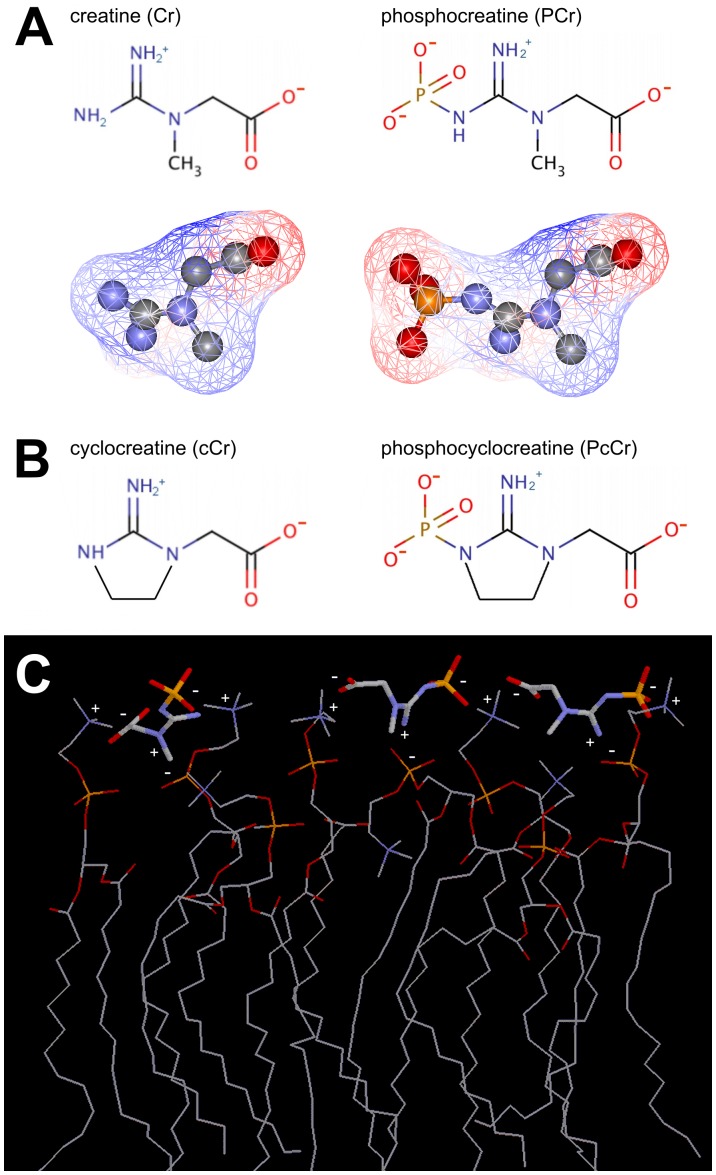
Structure and putative phospholipids interaction mechanism of phosphocreatine and its analogues. (A) Chemical structures of Cr, PCr (top) as well as the electrostatic potential (red, positive; blue, negative) at the solvent-accessible surface (mesh representation), superimposed on the ball-and-stick representations of Cr and PCr (bottom). (B) Chemical structures of the cyclic analogues cCr and PcCr. (C) Proposed interaction between PCr (bold line representation) and the zwitterionic headgroups of an array of eight dioleoyl phosphatidylcholine molecules (DOPC, thin line representation) with (partial) charges indicated. Structures prepared with WebLabViewer Pro v4.0 (for details see Materials and Methods).

The PCr/phospholipid interaction depends on polar interactions, since the phospho-moiety seems to be important for membrane binding and increasing ionic strength diminished the interaction in SPR experiments (not shown). In fact, many phospholipids are zwitterions, with their head group exposing at least one negative phosphate and one positive nitrogen-containing group. Also all examined guanidino compounds have a zwitterionic character due to their guanidino and carboxyl groups, and an additional full negative charge is present in the phosphorylated compounds (Fig. 7A,B). Interestingly, another zwitterion, taurine, has repeatedly been reported to interact with phospholiposomes [66] or cell membranes [67]. With their alternating positive and negative surface potential (Fig. 7A), these compounds are well suited to intercalate between and crosslink the zwitterionic phospholipid head groups, with the larger phosphorylated compounds carrying three different charged surfaces being more efficient. Indeed, the molecular dimensions of PCr fit very well to the distances of head groups in a planar PC membrane of 6–10 Å, allowing for numerous polar interactions (Fig. 7C). This would explain the enhanced phospholipid packing suggested by increased lipid phase transition temperature. However, additional binding determinants may contribute to the observed membrane stabilizing effect, such as sterical accessibility, distance of polar groups in the lipids [68], cooperative phenomena, or the initial fluidity of the membrane.

Changes in the properties of lipid bilayers induced by PCr binding were very clearly indicated by our ^31^P-NMR and DSC *in vitro* experiments, as well as in cell-based RBC protection assays. ^31^P-NMR detected a distortion in the lineshape of the ^31^P-NMR signal upon addition of PCr (and much less so Cr). Several examples of such alteration of the powder spectrum have been reported in literature and have been attributed to a preferential, magnetically-induced orientation of the bilayer with the normal perpendicular to the magnetic field which results in the deformation of the spherical liposomes to prolate ellipsoids [59], [69]–[71]. The macroscopical magnetic orientation observed in phospholipids in magnetic fields is due to the anisotropy of their magnetic susceptibility [70]. Generally, the corresponding energy contribution is smaller than the thermal energy and the membrane curvature energy and does not affect the axially symmetric ^31^P NMR powder pattern. On the other side, systems where the magnetic energy is large will show a preferential orientation of lipid bilayers. Factors which affect the extent of the magnetically induced orientation are, for instance, the strength of the static magnetic field, the size of the liposomes, the bulk viscosity, the bending elastic energy of the membrane, and the anisotropy of the magnetic susceptibility of the phospholipids upon binding of lipophilic molecules [70]. The addition of PCr (and possibly Cr) could induce a preferential orientation of the lipid vesicles for instance, by affecting the elasticity of the membrane, the size of the liposomes, or the total anisotropy of the magnetic susceptibility of the metabolite-lipid system. The influence of PCr on liposome shape as indicated by NMR could also explain the relatively strong SPR signal, because such a change could add to the signal due to metabolite binding to the liposome surface.

Direct evidence for altered membrane properties induced by PCr binding was provided by DSC. PCr binding led to a small but specific and significant shift in the phase transition temperature of a model lipid, corroborating the hypothesis of enhanced membrane packing. This interpretation is also consistent with earlier data on increased structural order in PCr-treated cardiac sarcolemmal preparations [49] or motional restriction of Cr and PCr in muscle [51]. Physiologically relevant effects of PCr/phospholipid interaction were evidenced by experiments with LUVs and red blood cells (RBC). PCr efficiently protected membranes and cells against lysis induced by a membrane permeabilizing peptide, a mild detergent, oxidative or hypoosmotic damage. Thus, the more tight packing of membrane lipids or even the presence of PCr itself protects sensitive lipid residues from different kinds of damaging agents, in particular from oxidation. The fact that under most conditions the protective effect of PCr is moderate does not diminish its potential *in vivo* significance.

Identification of mainly PCr (and PcCr) as interactors with phospholipids gives new mechanistic insight into the biological action of these guanidino compounds. First, it suggests that the presence of active CK is essential to generate the more potent PCr from Cr *in vivo*. This is also true for nutritional supplementation or intravenous injection, since PCr is prone to hydrolysis at acidic pH and does not represent a substrate for cellular uptake by the Cr transporter [17]. Second, and most importantly, phospholipid binding of PCr, Cr or their cyclic analogues could account for protective effects that are not exclusively related to high-energy phosphoryl transfer and the cellular energy state. An important example is the protection by Cr from oxidative stress, which induces harmful modifications of various biomolecules, including membrane phospholipids, in many pathological states like ischemia/reperfusion injury in heart or brain, or in acute and chronic heart failure [72], [73], neurodegenerative and neuromuscular diseases [74], [75], as well as in aging [76]. Protective effects of Cr have been reported for all these pathologies (for review see [1]–[5]), and depletion of intracellular PCr often correlated with membrane destabilization and destruction [47], [77]. In particular in ischemic myocardium PCr has been shown to inhibit process of sarcolemmal phospholipid degradation to lysophosphoglycerides [78]. Thus, favouring higher PCr levels would be beneficial for both, cell energetics and membrane structure. Since extracellular PCr is unlikely to transverse plasma membranes but obviously can bind to and interact with the outer leaflet of plasma membranes, our data explain the enigmatic fact that externally applied PCr in cardioplegic solutions displayed significant cardiac protection [79]–[81]. Other examples are the anti-tumor and neuroprotective activities found in different experimental systems for all guanidino compounds tested here, cCr or PcCr, as well as Cr or PCr [26], [29], [31], [33], although cCr and PcCr are unlikely to improve cellular energetics. As with PCr, the accumulating PcCr seems to be the active compound, since e.g. CK activity is mostly necessary for the inhibitory effect of cCr on tumor growth [26], [27].

The direct interaction of these guanidino compounds with membrane phospholipids and the resulting modulation of membrane properties, including phospholipid packing and membrane stabilization, could not only have protective effects in pathological situations, but may also play a role under normal physiological conditions where membrane-bound PCr may be important for membrane topology or membrane-related processes. Lipid interaction of PCr, leading to specific pools of membrane bound metabolites (see [82]), could also play a role in the organization and efficiency of processes in cellular energy transduction and metabolite channelling, which are largely membrane bound.
